# Peri-operative, functional and early oncologic outcomes of salvage robotic-assisted radical prostatectomy after high-intensity focused ultrasound partial ablation

**DOI:** 10.1186/s12894-020-00656-9

**Published:** 2020-07-01

**Authors:** James E. Thompson, Ashwin N. Sridhar, Greg Shaw, Prabhakar Rajan, Anna Mohammed, Timothy P. Briggs, Senthil Nathan, John D. Kelly, Prasanna Sooriakumaran

**Affiliations:** 1 Department of Uro-oncology, University College London Hospital, London, W1G 8PH UK; 2 Faculty of Medicine, University of New South Wales, Kensington, Australia; 3 Division of Surgery & Interventional Sciences, University College London, London, UK; 4 Centre for Molecular Oncology, Barts Cancer Institute, Queen Mary University of London, London, UK; 5 Nuffield Department of Surgical Sciences, University of Oxford, Oxford, UK

**Keywords:** Prostate cancer, HIFU, Salvage therapy, Radical prostatectomy, Robotics

## Abstract

**Background:**

Partial ablation of the prostate using high-intensity focussed ultrasound (HIFU-PA) is a treatment option for localised prostate cancer. When local recurrence occurs, salvage robot-assisted radical prostatectomy is a treatment option for selected patients, but there is a paucity of data on the peri-operative safety, functional and oncologic outcomes of sRARP..

The objective of this study was therefore to describe peri-operative safety, functional and early oncologic outcomes following salvage robot-assisted radical prostatectomy (sRARP) for local recurrence after HIFU-PA.

**Methods:**

Retrospective analysis of a prospective database of 53 consecutive men who underwent sRARP after HIFU-PA from 2012 to 2018. Continence and erectile-function were reported pre-HIFU, pre-sRARP, 3-months post-sRARP and 12-months post-sRARP. Complications, PSMs and need for subsequent ADT/radiotherapy were assessed.

**Results:**

45 men were suitable for inclusion and had sufficient data for analyses. Median duration from HIFU to sRARP was 30.0 months and median follow-up post-sRARP was 17.7 months. Median age, PSA and ISUP group were 63.0 yrs., 7.2 ng/mL and 2; 88.9% were cT2.

Median operative-console time, blood loss and hospital stay were 140 min, 200 ml and 1 day respectively. Clavien-Dindo grade 1, 2 and 3 complications < 90 days occurred in 8.9, 6.7 and 2.2%; late (>90d) complications occurred in 13.2%.

At sRARP pathology, ISUP 3–5 occurred in 51.1%, pT3a/b in 64.5%, and PSMs in 44.4% (37.5% for pT2, 48.3% for pT3).

Of men with > 3-months follow-up after sRARP, 26.3% underwent adjuvant radiotherapy/ADT for residual disease or adverse pathologic features; 5.3% experienced BCR requiring salvage ADT/radiotherapy. Freedom from ADT/radiotherapy was 66.7% at 12-months.

Pad-free rates were 100% pre-HIFU, 95.3% post-HIFU, 29.4% 3-months post-sRARP, and 65.5% 12-months post-sRARP. Median IIEF-5 scores pre-HIFU, post-HIFU, 3- and 12-months post-sRARP were 23.5, 16, 5 and 5, respectively. Potency rates were 81.8, 65.5, 0 and 0%, respectively. Bilateral/unilateral nerve sparing were feasible in 7%/22%.

**Conclusion:**

Salvage RARP was safe with acceptable but sub-optimal continence and poor sexual-function and poor oncologic outcomes. One in three men required additional treatment within 12-months. This information may aid men and urologists with treatment selection and counselling regarding primary HIFU-PA vs primary RARP and when considering salvage RARP.

## Background

Partial Ablation (PA) is proposed as a less morbid alternative to radical therapy for localized prostate cancer [1]. High-intensity focused ultrasound (HIFU) is one of several ablative modalities that have been approved for clinical use within prospective registry studies in the UK [2], although it remains experimental according to international guidelines [3, 4]. Evidence of oncologic efficacy for HIFU-PA is limited to observational cohorts with short follow-up, using PSA or radical treatment-free survival endpoints and without comparison to a matched group undergoing radical treatment [1, 5, 6].

Prostate cancer is often multi-focal and thus local recurrence after PA is common; 37–41% experienced recurrence requiring further treatment within 5 years and more than half (54%) by 8-years in two large, multi-centre series [6, 7], despite the fact that only a third underwent biopsy after HIFU; the rate of recurrence may therefore increase with longer follow-up.

There is very little published data on outcomes for salvage RARP after HIFU-PA. sRARP after radiotherapy is associated with adverse pathologic features, high PSM rates, low continence rates, poor erectile function and higher anastomotic leak/stricture rates compared to primary RARP [8–10]. After PA, Nunes-Silva et al. reported similar continence but worse sexual function and higher recurrence rates for sRARP compared to primary RARP in a matched-pair analysis [11]. Marconi et al. recently reported on their cohort of 82 men undergoing sRARP after FA by two surgeons in which they demonstrated safety and feasibility as well as impressive 83% pad-free continence at 1 year; however, potency was low (14% at 1 year) and the incidence of BCR was high (64% at 3-years) [12].

The most commonly used PA modality in the UK is HIFU and yet there are presently no published cohorts reporting on sRARP purely after HIFU-PA. This limits treatment selection and pre-treatment counselling for men with local recurrence after HIFU-PA or considering HIFU-PA as primary therapy.

We recently reported detailed pathologic outcomes in a series of 35 men and found high rates of multifocal cancer, Gleason upgrading, locally advanced (pT3a/b) cancer and positive margins, with a high rate of pT3 recurrence in-field and most PSMs being in-field due to the challenges of dissecting microscopic T3 cancer when it was densely fibrotic to the neurovascular bundles, pelvic floor muscles and rectum [13]. The primary objective of the present study was therefore to describe peri-operative, oncologic and functional outcomes of sRARP following HIFU-PA.

## Methods

### Study design and participants

Shortly after commencing sRARP procedures for recurrence post-HIFU, the Department of Pelvic Uro-Oncology at UCLH established a prospectively maintained electronic database to audit and analyse safety, oncologic and functional outcomes of sRARP post-HIFU. Data is collected and entered in real-time by a team of clinical nurse specialists and research fellows, using a combination of medical records, pathology reports and validated questionnaires. This is the first comprehensive analysis reporting all of these outcomes from this database.

53 men underwent sRARP after focal therapy from January 2012–August 2017 at our institution. Data on the exact number of men who underwent focal HIFU up until 2017 was not available to the authors to ascertain a precise estimate of recurrence, but a recent study from two institutions (including ours) reported on outcomes for 1032 men who underwent focal HIFU over 2005–2017 [7]. Some of the men in that cohort study underwent salvage RARP at other institutions for which the results of sRARP are published elsewhere [12]; therefore the aforementioned cohort study is better able to assess the incidence of failure and salvage treatment than the present cohort.

We excluded 8 men, because including men with bilateral cancer (3 whole-gland HIFU) and ablative technologies that have dissimilar tissue effects (5 had other ablative modalities, i.e. cryotherapy or RFA) may otherwise have confounded outcomes. Median (IQR) follow-up was 17 months (IQR 15.4–20.5) after sRARP and 46.4 months (IQR 33.6–68.6) after HIFU. 33 men had ≥12-months follow-up.

### Eligibility/selection for HIFU

All men had MRI and biopsy confirming prostate cancer and were deemed suitable for PA via MDT; most were ‘ideal’ candidates in line with prospective study protocols [14] or expert consensus criteria [15, 16]: PSA ≤15 ng/mL; ISUP grade group 1–3; cT2; uni-focal on MRI and biopsy; all were considered suitable for HIFU-PA at the time of treatment. On retrospective evaluation by the focal therapy team, however, 19/45 were not ‘ideal’ or received sub-optimal treatment according to (i) subsequently refined selection criteria or (ii) anatomic relative contra-indications (e.g. large gland, anterior/ apical location, capsular abutment suspicious for EPE) or (iii) technical treatment problems (e.g. probe problems, prominent calcifications, suboptimal Uchida changes suggesting incomplete ablation).

### Interventions and follow-up post-HIFU

All HIFUs were performed as part of UK-wide registry or NCRI studies [2] using the *Sonoblat*e 500® device (Sonacare Medical, North Carolina). Our monitoring protocol following HIFU was described previously [14], including 3-monthly PSA, 6–12 month MRI and targeted/ template biopsy for abnormal MRI.

### Selection criteria for sRARP

Given this is a retrospective analysis, no prospective selection criteria were defined. General selection criteria for sRARP were:

- Unsuitable for redo FA (e.g. bilateral/ high-risk cancer) or preference towards radical treatment;
- Age < 75yo and fit for major surgery;
- T1-3aN0M0, surgically resectable on MRI and DRE;
- Accepting of risks and side effects of surgery.

Pre-operative imaging included pre-biopsy MRI routinely, then bone scan and/or Ga68-PSMA PET CT for high-risk patients.

Salvage RARPs were performed by experienced surgeons (> 250 primary RARPs); biopsies and sRARP histology was reported by sub-specialist uro-pathologists using 5 mm axial step-sectioning with apical and basal shaves.

### Outcomes evaluated

Clinical, imaging and pathologic characteristics (pre-HIFU, pre-sRARP, and post-sRARP) were collected. PSM rates were reported overall and stratified by pT-stage, focality (single vs multifocal), length (< 3 vs ≥3 mm) and relation to ablation zone (in-field versus out-of-field). Clinically ‘significant’ margin was defined as pT3 PSM or ≥ 3 mm length or multifocal.

For post-sRARP oncologic outcomes:

(i) Residual biochemical disease was defined as PSA ≥0.2 ng/mL and rising on successive measurements without postoperative PSA nadir < 0.2 ng/ml;
(ii) BCR was defined as no residual biochemical disease followed by a PSA ≥0.2 ng/mL and rising on 2 successive measurements.
(iii) Adjuvant treatment was defined as initiation of ADT/RT for high-risk pathologic features or residual biochemical disease;
(iv) Salvage treatment was defined as initiation of ADT/RT for BCR.

Peri-operative parameters were extracted from operation reports and discharge summaries. Complications were attained from discharge summaries, clinic letters, emergency department records and urology nurse phone calls after sRARP. Complications were classified using the Clavien grading system as early (< 90 days) or late (90–365 days).

Continence and sexual function data were extracted from prospective questionnaires by independent researchers/nurse specialists. Pre- and post-HIFU-PA, leak-free and pad-free status were assessed via EPIC-QOL. After sRARP at 3- and 12-months, continence outcomes (number of pads/24 h) were assessed via ICIQ-SF. Primary sexual function variables were IIEF-5 score (5–25) and Q2 of the EPIC/IIEF sexual function domain, i.e. “how often are erections firm enough for sexual intercourse” (1–5: 1 = none, 3 = half, 5 = all of the time). Q2 responses 3–5 were defined as potent for binary endpoint analysis. PDE5 inhibitors were permitted but men requiring invasive erectile aids were classified as impotent.

## Results

### CLINICO-PATHOLOGIC CHARACTERISTICS (pre-HIFU, pre- and post-sRARP)

Clinico-pathologic characteristics pre-HIFU, pre-sRARP and post-sRARP are shown in Table 1 including age, PSA, Gleason sum, cancer volume and cT-stage.

**Table 1:** – Comparative clinico-pathologic characteristics pre-HIFU, pre-sRARP and post-sRARP (*n* = 45)

Variables	Pre-HIFU	Pre-sRARP	Final sRARP pathology
Age, years (IQR)	62 (IQR 60–66)	63.0 yrs. (IQR 61–66)	–
PSA, median (IQR)	7.3 (5.5–8.9)	6 (3.9–10.0)	–
Gleason score, n (%):			
3 + 3	9 (20.0)	3 (6.7)	0 (0)
3 + 4	29 (64.4)	22 (48.9)	22 (48.9)
4 + 3 (or 3 + 4 with tertiary grade 5)	5 (11.1)	15 (33.3)	18 (40.0)
4 + 4 (or 4 + 3 with tertiary grade 5)	2 (4.4)	2 (4.4)	1 (2.2)
4 + 5	–	3 (6.7)	4 (8.9)
T-stage*, n (%):			
T2	43 (95.6)	40 (88.9)	16 (35.6)
T3a	2 (4.4)	4 (8.9)	21 (46.7)
T3b	–	1 (2.2)	8 (17.8)
Max cancer core length in mm, median (IQR)	7 (5–10)	6 (3–8)	n/a
Number of cores taken, median (IQR)	36 (14–58)	13 (8–24)	n/a
Percentage of positive cores, median (IQR)	17 (11–30)	30 (14–40)	n/a

HIFU treatment details are shown in Table 2 including the proportion who were ideal in retrospect according to consensus selection criteria, type of biopsy pre-HIFU the anatomic extent of the ablation field in each patient, the number of treatments and presence of low-grade cancer left untreated.

**Table 2:** – HIFU treatment technical details (n = 45)

Ideal according to consensus criteria, n (%)	26 (57.8)
Prostate volume, median (IQR)	35 (27–46)
Location of treatment (combined treatment fields for *n* = 5 with 2 HIFU treatments)	
Hemi-gland unilateral	
Hemi-gland with extension across midline or into SV	16
Hemi-gland anterior	7
Hemi-gland posterior	1
Quadrant (e.g. unilateral posterior)	1
Focal ablation (eg posterior right basal segment)	13
Subtotal (extended hemi-ablation, sparing lateral aspect of contralateral side)	6
	1
Number of HIFU treatments	
1	37
2	8
Known ‘insignificant’ cancer left untreated at HIFU	
Yes	21 (47.7)
No	23 (52.3)
Type of biopsy pre-HIFU:	
TTMB TP 5 mm Mapping + MRI-Targeted (if targets)	24 (53.3)
12–20 core TRUS + MR-targeted (if targets)	17 (37.8)
Targeted alone	3 (6.7)
Not documented	1 (2.2)

Median time from diagnosis to sRARP was 43-months (IQR 30–67); median time from last HIFU to sRARP was 30-months (IQR 18–63).

#### PERI-operative outcomes and complications

Peri-operative outcomes and complications are shown in Table 3. Median (IQR) operative-console duration was 140 min (120–180). Median (IQR) blood loss was 200 ml (100–300). Median (IQR) length of stay was 1 day (1–2). The incidence of early (< 90 days) Grade 1, 2 and 3 complications were 8.9, 6.7 and 2.2%, respectively; the incidence of late (90–365 days) post-operative complications requiring re-admission was 15.2% (Table 3).

**Table 3:** - Early and Late complications after sRARP according to Clavien group (n = 45)

Early complications (< 90 days)	Number (%)	Description
Grade I	4 (8.9%)	(i) 1x AKI (self-limiting)
		(ii) 3 asymptomatic leaks on initial cystogram requiring prolonged catheterisation
Grade 2	3 (6.7%)	(i) 1x UTI 2 weeks post-op requiring oral antibiotics;
		(ii) 1x readmission for anastomotic leak and fever requiring IV antibiotics and observation (no intervention)
		(ii) 1x transfusion for retroperitoneal bleeding (did not require surgical/ radiologic intervention)
Grade 3a Grade 3b	1 (2.2%)	3b: Laparotomy, evacuation of clot and re-fashinoing of vesico-urethral anastomosis for haematoma causing anastomotic leak/ disruption
Grade 4	0	–
Grade 5 (Death)	0	–
**Total**	**8/45 (17.8%)**	–
Late complications (90 days – 12 months)*	5/33 (15.2%)	(i) 3x bladder neck contractures requiring 1 or more cystoscopy + optical dilation
		(ii) 1x Hemolock clip protruding into anastomosis causing LUTS
		(iii) 1x Small bowel obstruction (resolved with conservative management) due to adhesions in the same man who underwent laparotomy < 90 days.

*Note to Table: All 45 men completed 90-day peri-operative outcome follow-up; 12 men have not yet reached 12-months follow-up and therefore the sample size is *n* = 33

#### Oncologic outcomes

The overall (clinically significant) PSM rate was 44.4% (26.3%), with 81% PSMs being in-field. The pT2 overall (clinically significant) margin rate was 37.5% (12.5%), with 83.3% PSMs being in-field. The pT3 positive margin rate was 48.3%, with 85.7% PSMs being in-field.

HIFU-PA did not appear to compromise oncologic outcomes in the out-of-field regions. The rate of out-of-field (OOF) PSMs was very low, i.e. 6% for pT2 (17% OOF of 37.5% with PSM) and 7% for pT3 (14% OOF of 48% with PSM). This suggests that the effects of HIFU-PA on tissues away from the ablation zone were limited, such that surgeons were able to perform precise surgical dissection and also this suggests that tumour volumes may have been low in OOF regions, further reducing the probability of a PSM.

38 men had 3-month post-RP PSA data; 15.8% of men had residual biochemical disease (BCD) requiring immediate adjuvant ADT/RT; and additional 5.3% (2/38) received early adjuvant ADT + RT due to high risk features (both pT3a/b + ISUP 3–5 + PSM) and the remainder were managed expectantly; 10.5% (4/38) experienced BCR requiring salvage ADT/RT (three within 12 months, one at 18 months). Of men with minimum 12-month follow-up or having reached the ADT/RT event endpoint, 66.7% (22/33) were free from BCR and any adjuvant/salvage therapy at 12 months and 63.6% (21/33) at last follow-up (median 18-months). No men experienced local/distant clinical recurrence or died during follow-up.

#### Continence outcomes

44/45 men had 3-month pad-free status documented (*n* = 1 with missing data due to failure to attend follow-up). 2 additional men were excluded from post-sRARP analysis due to using pads pre-sRARP for urge incontinence. At 3 (12) months post-sRARP, 33.3% (65.5%) were pad-free and 61.9% (86.2%) were socially continent, respectively. 3 & 12-month median (IQR) ICIQ-scores were 4 (IQR 0–9) and 0 (0–3), respectively.

#### Feasibility of nerve sparing at SRARP post-HIFU

At sRARP, unilateral (bilateral) nerve sparing was feasible in 22.2% (6.7%); 11.1% of all NVBs (45 men × 2 NVBs per patient) were preserved. Nerve sparing was not feasible due to HIFU-induced NVB fibrosis in 60.8%, suspicion of posterolateral EPE on MRI or high-grade cancer near the NVB in 23.0% and erectile dysfunction in 17.9%.

#### Sexual function outcomes

90.9% (40/44) were potent pre-HIFU based on Q2 EPIC, IIEF-5 and/or clinical notes documentation of erectile-function; 73.0% were potent post-HIFU. No man (0/31) recovered erectile-function at 3 or 12-months post-sRARP

#### PENTAFECTA outcomes in men with at least 12-months follow-up

No man achieved the composite pentafecta [17] outcome, defined as all of: (i) potent adequate for intercourse half the time or more with/ without PDE5Is; (ii) continent not requiring any daily pad use; (iii) free from BCR; (iv) negative surgical margins; and (v) free from early or late peri-operative complications. 8/29 (27.6%) achieved optimal outcomes across the four domains excluding potency.

#### Descriptive comparison to primary RARP outcomes

Table 4 compares our published primary RARP outcomes [18, 19] to our salvage RARP outcomes detailed herein.

**Table 4:** Summary of primary versus salvage RARP outcomes at our institution

Baseline or Outcome Variable	Primary RARP(16, 17)	Salvage RARP
Complication rate (Clavien-Dindo grade) (%)		
Early Grade 1–3	7–13	17.8
Early Grade 4	0.4*	0
Early Grade 5	0*	0
Anastomotic leak on cystogram	2*	11.1
Late bladder neck contracture/ clip	0.5*	10.5
Pre-RARP D’Amico risk group (%)		
Low	3	6.7
Intermediate	36	73.3
High	61	20.0
Pathologic T-stage (%)		
pT2	53	35.5
pT3	47	64.5
pT3a	33	46.6
pT3b	14	17.8
Positive surgical margin rate (%)		
Overall	17.3	44.4
pT2	9.6	37.5
pT3	26.1	48.3
Continence		
Pad-free at 3-months	67	33.3
Pad-free at 12-months	85.4	65.5
Socially continent at 12-mo (0–1 pad)	89.2	86.2
Proportion where nerve-sparing (NS) feasible		
Feasibility of bilateral NS (%)	18	6.7
Feasibility of unilateral NS (%)	34	22.2
Feasibility of bilateral NS in high-risk Ca	10	0.0
Proportion who received bilateral nerve-sparing and were potent at 12-months (potent pre-RARP)#	70	0 (0/2)

*Institutional audit data from latest institutional audit for calendar year 2017, *n* = 605 primary RARPs; #defined as erections adequate for intercourse at least half the time with or without the aid of PDE5Is

## Discussion

To our knowledge, this is the second largest reported cohort of sRARP after PA and the first report on clinical outcomes purely using HIFU-PA as the ablative modality (Marconi et al. and Nunes-Silva et al. included a variety of PA ablative techniques). We found that sRARP is technically feasible, reasonably safe, and has acceptable continence outcomes long-term. However, erectile-function recovery and oncologic results were poorer than expected for a cohort whom started out with favourable disease, and continence outcomes are arguably not ideal. These results are consistent with the recent report by Marconi et al [12], with the exception that in the present study the continence rate was lower (66% vs 83%) and the significant PSM rate was higher (26% vs 13%), possibly as a result of a greater proportion of focal re-treatments pre-sRARP in our series.

In this cohort study, sRARP had acceptable perioperative outcomes overall, but with higher than expected rates of anastomotic leaks and bladder neck contractures; this may be due to HIFU-induced tissue damage to the bladder neck and/or urethra in some cases. A recent report suggests that tissue-scaffold bio-grafts may help reduce the risk of leaks in salvage cases [20]. The apparently worse continence outcomes compared to primary RARP in our series, also seem unfavourable compared to the report from Nunes-Silva et al [11], possibly due to unmeasured differences in patient characteristics, extent of PA tissue damage or sRARP surgical technique. Further studies will provide a larger evidence base for continence outcomes of sRARP following HIFU, thus we are participating in a multi-institutional study (RAFT, NIH trial NCT03011606).

Sexual function outcomes in our series were poor, with bilateral nerve sparing rarely feasible due to a combination of post-HIFU fibrosis, cancer factors and erectile dysfunction. Potency preservation cannot thus be a realistic aim of sRARP. The PSM rate was high in this cohort (44.4% overall, 37.5% pT2 and 48.3% pT3) despite wide excision at the site of previous HIFU and any high-grade or capsular tumor; this compares unfavorably to a recent report for primary RARP from our institution (17.3% overall, 9.6% pT2 and 26.1% pT3). Likewise, in a meta-analysis of over 400,000 cases, the pT2 and pT3 PSM rates were 11 and 37% [21]. PSM rates for sRARP after HIFU-PA were similar to salvage post-radiation [22], representing technical difficulty dissecting post-HIFU tissue planes with severe fibrosis/adherence to the rectum and/or pelvic floor after HIFU.

Early oncologic outcomes were worse than expected for a selected population who at baseline mostly had unifocal, intermediate risk, cT2 disease. 1 in 3 men required further salvage therapy within 12 months. This is consistent with the matched-pair analysis of salvage versus primary RARP by Nunes-Silva et al. and the Marconi et al. cohort, which both reported high BCR rates in salvage cohorts. It might arguably be best to therefore perform wide bilateral excision in all sRARP cases after PA in an attempt to gain better oncologic control, especially as the potency recovery in these men is poor. The poor outcomes of sRARP vs primary RARP need to be balanced against the fact that in the short-term, propensity score-matched analysis suggest primary HIFU-PA has superior continence and potency outcomes to primary RARP, albeit with a significant risk of requiring salvage treatment [23, 24].

Our oncologic results were similar to sRARP following radiotherapy where one-third experience BCR within 12 months [25], half by 5 years [22] and almost two-thirds by 10 years [26]. It must be stressed, however, that these salvage RARP cohorts represent a minority of all primary HIFU cases and this study did not aim to estimate the incidence of failure after primary HIFU. As a guide, approximately 1000 focal HIFUs have been performed at UCLH between 2005 and 2017; 50 sRARPs were performed at UCLH, 20 elsewhere (via the RAFT study), 50 underwent radiotherapy at UCLH (others elsewhere), some received watchful waiting/ ADT and ~ 20% were re-treated with HIFU. Therefore, whilst quantifying the recurrence rate is difficult outside of prospective studies, we estimate 35–40%, consistent with 37–41% reported by Guillaumier et al. and Stabile et al., in which 12% required radical treatment/ADT and another 25% received redo HIFU within 5 years [6], and 20% required radical therapy within 8 years [7].

Longer-term follow-up data is only available for whole gland HIFU; in two multi-centre registry study including cases from our institution, 37–41% required further therapy by 5 years and 54% by 8 years [7, 27], while another study reported 60% recurrence for intermediate-risk and 100% recurrence for high-risk disease by 10 years [28]. These studies suggest that the long-term (10–15 year) risk of requiring radical therapy after HIFU may be higher than short-term studies suggest. Hence, we advise caution in patient selection and counselling until long-term follow-up data are available for men undergoing PA, especially for those at higher risk of recurrence e.g. ISUP group 3–5, younger age, family history, or Afro-Caribbean ethnicity. Our findings that the majority of recurrences were in-field and apical/ anterior suggest that other relative contra-indications may include apical or anterior tumours and large glands. Finally, any indication of sub-optimal ablation at the time of treatment (e.g. probe issues, anatomic issues, heavy calcification, reduced Uchida changes, inadequate treatment effects on post-HIFU MRI, etc. should warrant early re-biopsy and consideration of early salvage radical treatment.

This study has limitations. Data collection was prospective, but study design and analyses were retrospective. Non-standardized patient selection, pre-HIFU and post-HIFU biopsy techniques and selection for sRARP preclude from drawing conclusions about the efficacy of primary HIFU. Furthermore, this study cohort represents only a small subset of the overall HIFU cohort. Standardized validated questionnaires were used wherever possible to assess continence and erectile-function, but some men had missing baseline or follow-up questionnaires and thus binary endpoints using data from the medical case notes were relied upon. To minimize bias, we utilized only data that were collected independently by a third party (clinical nurse specialist or independent erectile dysfunction/incontinence clinic sub-specialist urologist) in our analyses. Finally, although we made comparisons to our primary RARP cohort to provide context to our results, these were unadjusted for baseline variables. Hence, further studies with matched-pair analyses of salvage versus primary RARP are required to validate our findings.

Men who choose HIFU-PA as initial therapy for their localized prostate cancer do so to avoid the adverse effects of radical therapy. Given the recurrence rates of HIFU-PA, it is important these men are adequately counselled on outcomes of salvage radical therapy should their disease recur. For a proportion of men considering this novel therapy, the standard-care option of radical prostatectomy may become preferable given the data herein. Further, with recent innovations in RARP such as Retzius-sparing [29], which has been shown to decrease functional compromise, the incremental functional benefits of HIFU-PA over RARP may be reduced.

Standard-care options of management for locally-recurrent prostate cancer in men after HIFU-PA are poorly defined, since HIFU-PA as initial therapy is not itself considered standard-care [3, 4]. Salvage options vary from systemic therapy to local radical therapy. sRARP is utilized by many high-volume expert RARP centres, and our data suggest that even in an experienced centre, overall outcomes are disappointing. Whether salvage radiotherapy provides better outcomes is unknown and is under investigation at our institution.

## Conclusions

Salvage RARP for recurrent prostate cancer after HIFU-PA is safe and feasible. Erectile recovery, continence recovery cancer recurrence and anastomotic stricture rates, however, appear worse than for primary RARP. HIFU-PA is an attractive choice to avoid the morbidity of radical treatment, but patients and their clinicians should consider the sub-optimal outcomes of sRARP when making initial and salvage treatment choices.

## Data Availability

De-identified raw datasets analysed for this study are available from the corresponding author on reasonable request, conditional on institutional data sharing agreements.

## Abbreviations

ADT: Androgren Deprivation Therapy
BCR: Biochemical Recurrence
EPE: Extra-Prostatic Extension
HIFU-PA: High-Intensity Focussed Ultrasound – Partial Ablation
ISUP: International Society of Uropathology
MRI: Magnetic Resonance Imaging
PCa: Prostate Cancer
PSM: Positive Surgical Margin
QOL: Quality of Life
RT: RadioTherapy
sRARP: Salvage Robotic-Assisted Radical Prostatectomy
UCLH: University College London Hospital
